# PPP2CB aggravates atherosclerosis-related dyslipidemia via LOX-1/MAPK/ERK signaling pathway

**DOI:** 10.1186/s12944-025-02647-x

**Published:** 2025-07-03

**Authors:** He An, Dong-liang Cheng, Xian-ru Xia, Xian-dong Li, Zhi-hua Ruan, Chun-yan Peng

**Affiliations:** 1 Department of Laboratory Medicine, Taihe Hospital, Hubei University of Medicine, Hubei Shiyan, China; 2 Cardio-Thoracic Surgery Department, Taihe Hospital, Hubei University of Medicine, Hubei Shiyan, China; 3 Department of Anesthesiology, Taihe Hospital, Hubei University of Medicine, Shiyan, Hubei Province China; 4 Hubei Key Laboratory of Embryonic Stem Cell Research, Hubei University of Medicine, Hubei Shiyan, China

**Keywords:** Dyslipidemia, Atherosclerosis, PPP2CB, LOX-1, Liver cells

## Abstract

**Background:**

Dyslipidemia has been extensively documented as a key driver of cardiovascular pathology. Regulating lipid homeostasis holds promise for treating atherosclerosis (AS). Although the protein phosphatase 2 catalytic subunit beta (PPP2CB) is involved in post-transcriptional gene regulation, its role in AS-associated dyslipidemia is not well understood.

**Methods:**

The study included both human participants and animal models. The following techniques were employed: cell culture, extraction of exosomes, preparation of pooled hyperlipidemic serum (HS), transfection, western blotting, immunofluorescence staining, quantitative reverse transcription polymerase chain reaction (qRT-PCR), co-immunoprecipitation, low-density lipoprotein cholesterol (LDL-C) uptake assay, biochemical assays, assessment of aortic atherosclerotic lesions, as well as statistical analysis.

**Results:**

This study identified a marked upregulation of PPP2CB expression in peripheral blood leukocytes of AS patients, artery plaque of ApoE^−/−^ mice given a high-fat diet, and hepatic cells exposed to hyperlipidemic stimuli. Overexpression of PPP2CB in hepatic cells exacerbated lipid accumulation and low-density lipoprotein uptake, whereas silencing PPP2CB mitigated this effect. Immunofluorescence co-localization and co-immunoprecipitation analysis confirmed a direct interaction between PPP2CB and lectin-like oxidized LDL receptor-1 (LOX-1). Notably, PPP2CB manipulation disrupted hyperlipidemia-induced LOX-1 expression. Additionally, PPP2CB-mediated lipid dysregulation was linked to the activation of the LOX-1/ mitogen-activated protein kinase (MAPK)/ extracellular signal-regulated kinase (ERK) signaling cascade.

**Conclusions:**

These results unveil PPP2CB as a novel lipid regulator in the progression of pathological AS and highlight its involvement in signaling regulation during abnormal lipid metabolism. PPP2CB could be considered a promising candidate for biomarker development and therapeutic intervention in AS.

**Supplementary Information:**

The online version contains supplementary material available at 10.1186/s12944-025-02647-x.

## Introduction

Cardiovascular disease remains the foremost cause of human mortality, as well as a significant contributor to the global disease burden, with its incidence having risen by 21.1% in the past decade [1, 2]. Atherosclerosis (AS), characterized by excessive lipid accumulation within the arterial intima [3], is central to the main pathological cascade. This process encompasses multiple pathological events, such as inflammatory responses, oxidative damage, as well as foam cell formation. An overload of lipids in the bloodstream enters the vascular lining, settles within the inner arterial layer, and subsequently initiates preliminary atherosclerotic lesions [4]. Considering the critical involvement of dyslipidemia during the initiation and advancement of AS, there is a need for a comprehensive understanding of the molecular pathways that regulate this process.

As the largest metabolic organ in the human body, the liver plays a crucial role in lipid metabolism by influencing fat synthesis, breakdown, and the uptake and secretion of serum-derived lipoproteins [5]. Extensive clinical and laboratory evidence suggests that disturbances in liver lipid regulation lead to increased free fatty acids and triglycerides, which affect low-density lipoprotein (LDL) production and transport, thereby promoting lipid deposition in the blood vessel walls [6, 7]. Inflammatory pathways mediated through reactive oxygen species (ROS) along with cytokines further exacerbate blood vessel inflammation and plaque formation, driving the progression of AS [8]. At the same time, hepatic lipid accumulation activates inflammatory responses, causing endothelial cell dysfunction and increasing the formation of oxidized LDL (ox-LDL), which accelerates plaque formation [9, 10]. Liver kinase B1 functions as a pivotal regulator in inhibiting lipid internalization by engaging with AMP-activated (AMPK) protein kinase, which helps to prevent vascular endothelial cell injury in AS [11]. Advanced glycation end products activate the RAGE/NF-κB pathway activity, stimulating LDL transcytosis and accelerating AS progression [12]. Under oxidized LDL (ox-LDL) stimulation, the interaction between DExH-Box helicase 9 and p65 enhances inflammation in macrophages, thereby promoting AS progression [13]. However, the specific molecular mechanism linking hepatic lipid metabolism disturbance and AS is not yet fully understood.

Phosphorylation of proteins represents a highly adaptable and reversible process, with the phosphorylation state being controlled through antagonistic actions of kinases and phosphatases. Multiple studies have suggested that serine/threonine phosphorylation can serve as on/off switches in various metabolic disorders [14] and is also closely related to the occurrence and development of AS [15]. Protein phosphatase 2 A (PP2A), classified as a serine/threonine-specific phosphatase, contributes to the attenuation of LDL-induced endothelial dysfunction and foam cell formation, thereby preventing the progression of AS [16]. PP2A consists of three distinct subunits, including a structural subunit “A” (PP2A-A), a regulatory “B” subunit, and a catalytic component “C”. Its β isoform within the catalytic unit is known as protein phosphatase 2 catalytic subunit beta (PPP2CB) [17]. Previous research has indicated that PPP2CB is involved in controlling T-cell activation triggered by phorbol myristate acetate and ionomycin [18], associated with Parkinson’s disease pathogenesis [19], in addition to serving as an independent prognostic factor for bladder urothelial carcinoma [20]. Nevertheless, a direct association between PPP2CB and AS induced by lipid metabolism remains unreported in current studies.

Recent findings suggest a potential role for PPP2CB, a catalytic subunit of PP2A, in disease-related signaling, while its contribution to atherosclerosis is not yet been unknown [21, 22]. This investigation utilized high-throughput mRNA screening to identify elevated PPP2CB expression in plasma exosomes from individuals diagnosed with ST-segment elevation myocardial infarction (STEMI). This upregulation was further validated in a cohort of 308 patients with AS compared to 302 healthy controls.

Based on these observations, it was hypothesized that PPP2CB promotes hepatic lipid accumulation and contributes to atherosclerosis by interacting with LOX-1 receptor and activating the MAPK/ERK cascade. To test this hypothesis, a series of in vivo and in vitro experiments were conducted to elucidate the potential mechanistic role of PPP2CB in the development of atherosclerosis.

## Materials and methods

### Study participants

In June 2018, plasma exosome samples were collected from five patients with STEMI whose coronary angiography showed obstruction, as well as from three volunteers with negative coronary angiography, for high-throughput screening of mRNA chips. All sample collections were conducted at Taihe Hospital, Hubei University of Medicine. From June 2019 to September 2020, peripheral blood plasma was collected from 308 patients with AS and 302 healthy controls at the Department of Cardiology, Taihe Hospital, Hubei University of Medicine. All participants underwent coronary angiography. Patients who had acute heart failure, congenital heart disease, severe non-coronary cardiovascular disease, systemic infection, or inflammatory disease were excluded. In compliance with the Declaration of Helsinki, this research received ethical clearance from the Ethics Committee of Taihe Hospital. All enrolled subjects signed informed consent forms.

### Experimental animals

Five-week-old female ApoE (apolipoprotein E)^−/−^ mice (*n* = 12) were obtained from Biocytogen (Nanjing, China), then kept under a 12- h alternating light/dark cycle, with access to food and water in an environmentally controlled room (24 ± 2 °C and 60% humidity). Six mice were fed a normal chow diet (ND), and the remaining six received a high-fat diet (HFD) containing 21% fat and 0.3% cholesterol. After 12 weeks, all mice were euthanized, and their heart tissues were collected for PPP2CB expression analysis. The animal experimental procedures complied with the Guide for the Care and Use of Laboratory Animals (National Institutes of Health, Bethesda, MD, USA), and received approval from the Animal Care and Use Committee of Hubei University of Medicine.

### Cell culture

The HepG2, Huh7, and LO2 cell lines were obtained from the American Type Culture Collection (ATCC, Manassas, VA, USA). These cell lines were selected for their relevance to atherosclerosis and lipid metabolism research: HepG2 cells were used to study lipid metabolism and lipoprotein synthesis; Huh7 cells were used to investigate lipid accumulation and metabolic disorders; and LO2 cells served as a model of normal hepatic function for comparison. All cell lines were cultured in Dulbecco’s Modified Eagle Medium (DMEM, Corning Inc, NY, USA) supplemented with 10% fetal bovine serum (FBS; Gibco, Grand Island, NY, USA), and were maintained at 37 °C in a humidified incubator with 5% CO₂ (Thermo Fisher Scientific, Waltham, MA, USA). Trypsin (Gibco, Grand Island, NY, USA) was used to detach cells when they reached confluency.

### Extraction of exosomes

Ribo™ Exosome Isolation Reagent (Ribo, Guangzhou, China) was used to isolate exosomes from plasma. Briefly, frozen plasma samples were thawed to room temperature, after which the extraction reagent was added in a 1:3 ratio and mixed thoroughly. The mixture was stirred until it became turbid and then placed in a refrigerator at 4 °C for 30 min. Subsequently, it was centrifuged at 15,000 × g for 45 min at 4 °C. Finally, the supernatant was discarded, and the pellet containing exosomes was washed and recentrifuged for downstream analysis.

### Preparation of pooled hyperlipidemic serum (HS)

Patients with hypercholesterolemia were recruited. These patients provided blood samples, which were pooled and filtered using a 0.22-µm filter (Millipore, Billerica, MA, USA). Biochemical and hormonal levels were quantified, with results presented in Supplementary Table S1. Hypercholesterolemia was characterized by total cholesterol (TC) ≥ 6.2 mmol/L and/or LDL cholesterol ≥ 4.1 mmol/L [23]. The methods of Xie et al. 2019 were followed [24].

### Transfection

The expRNA-PPP2CB, siRNA-PPP2CB, expRNA-LOX-1, and siRNA-LOX-1 were designed and synthesized by Hanbio (Shanghai, China). HepG2, Huh7, and LO2 were seeded in 6-well plates at 1 × 10⁵ cells per well. When the cell confluence approached 60%, the expRNA-PPP2CB, expRNA-LOX-1, siRNA-PPP2CB, and siRNA-LOX-1 were introduced into cells via Lipofectamine™ 2000 (Invitrogen, USA). After cultured for 6 h at 37 °C in a 5% CO₂ atmosphere, the old medium was replaced with fresh medium to maintain growth, and cells were maintained for an extra 24 h prior to collection for downstream analysis.

### Western blotting

For protein extraction, cells were incubated with modified RIPA buffer (Beyotime, Shanghai, China) supplemented with fresh protease inhibitors (Beyotime, Shanghai, China) on ice for 30 min. Then, centrifugation was performed at 12,000 × g for 10 min at 4 °C, after which supernatants were harvested. The bicinchoninic acid (BCA) assay (Biosharp, Hefei, China) was employed to quantify protein concentration. Next, protein samples were mixed with 5× SDS loading buffer and boiled at 100 °C for 10 min, resolved via SDS-PAGE, and electroblotted onto PVDF membranes (Millipore, Billerica, MA, USA). The membranes were blocked with 5% skimmed milk for 1.5 h at room temperature, then washed three times with TBS-T, and exposed overnight at 4 °C to primary antibodies against GAPDH (Proteintech, Rosemont, IL, USA), PPP2CB, and LOX-1 (Abcam, Cambridge, UK). The next day, the membranes underwent triple washes with TBS-T and were treated with secondary antibodies for 2 h at room temperature. Afterward, the membranes were washed three times and incubated with enhanced chemiluminescence (ECL) substrate (Millipore, Billerica, MA, USA). Protein visualization was accomplished using a gel documentation platform (BIO-RAD, Hercules, CA, USA). Protein expression alterations were quantified via ImageJ. The western blotting process was performed in triplicate for all proteins.

### Quantitative real-time PCR (qRT-PCR)

Total RNA was isolated with the aid of TRIzol solution (Tiangen Company, Beijing, China). To evaluate the purity and concentration of the extracted RNA, a Nanodrop 3000 spectrophotometer (Thermo Fisher, USA) was employed. Reverse transcription was carried out using 1 µg of RNA as a template, following the instructions provided with the reverse transcription kit (Vazyme, Nanjing, China). qRT-PCR was performed using ChamQ SYBR Green Master Mix (Vazyme, Nanjing, China) on a CFX Manager platform (Bio-Rad Laboratories, Hercules, CA, USA). Primers utilized are presented in Supplementary Table S2.

### Fluorescent immunostaining

A 20-min fixation with 4% paraformaldehyde (Biosharp, Hefei, China) was performed on cells plated into 24-well plates, after which they were permeabilized with 0.1% Triton X-100 (Beyotime, Shanghai, China). Following this, cells were subjected to a 30-min blocking step using 5% FBS at ambient temperature. PPP2CB and LOX-1 primary antibodies were then added and incubated at 4 °C for the night. Subsequent to washing, appropriate secondary antibodies were used (1:200) for 1 h. Staining with 4′,6-Diamidino-2-Phenylindole (DAPI, Biosharp, Hefei, China) was conducted for 15 min, and Dako mounting agent (Biosharp, Hefei, China) applied before image acquisition via confocal scanning (Olympus, FV3000RS, Tokyo, Japan).

### Oil Red O-Based lipid staining

A 20-min fixation step with Oil Red O reagent (Solarbio, Beijing, China) was performed on cells, after which they were washed three times in phosphate-buffered saline (PBS), treated with 60% isopropyl alcohol (Aladdin, China) for 20–30 s, and were stained using Oil Red O staining solution (Solarbio, Beijing, China). After staining, the cells were washed with 60% isopropyl alcohol (Aladdin, Shanghai, China) until the background became clear. This washing step was repeated 2–3 times, and the slices were then treated with Oil Red O solution for 60 s. Finally, a drop of distilled water was applied to immerse the cells, and images of positively stained (red) cells were captured using an optical microscope (Leica, Wetzlar, Germany).

### Co-immunoprecipitation

Co-immunoprecipitation was performed following the protocol described by Yan et al. 2019 [25]. HepG2 cells received 10% HS solution for 24 h. The cells were subsequently lysed using a RIPA buffer. The resulting whole cell lysates were then incubated with specific antibodies overnight at 4 °C, with A/G-agarose (Santa Cruz, Biotechnology, Dallas, TX, USA) used for immunocomplex retrieval. Finally, Western blotting analysis was performed.

### Biochemical analysis

Following a 24 h transfection of expRNA-PPP2CB, siRNA-PPP2CB, expRNA-LOX-1 and siRNA-LOX-1 (Hanbio, Shanghai, China), the cells were treated with 10% HS for another 12 h at 37 °C. Subsequently, the supernatant medium was carefully collected through centrifugation (300 × g, 5 min) to eliminate debris, and stored at − 80 °C for future evaluation. Lipid profiles, including high-density lipoprotein (HDL), low-density lipoprotein (LDL), total cholesterol (TC), and triglyceride (TG), were quantified via an autoanalyzer (Beckman Coulter, Miami, FL, USA).

### LDL-C uptake assay

The LDL-C uptake was determined according to the manufacturer’s protocol of LDL-DyLight™ 550 (Abcam, Cambridge, MA, UK). Briefly, HepG2, Huh7, and LO2 cells were seeded into 24-well plates and maintained in a medium supplemented with 20 nM PD98059 (Abcam, Cambridge, MA, UK) or vehicle alongside 10% FBS. After a 24 h treatment, media were changed to LDL-DyLight 550 (Abcam, Cambridge, MA, UK) working solution with 20 nM PD98059 or its vehicle. Following a 4 h incubation, cells were washed in PBS and imaged by fluorescence microscopy with excitation and emission wavelengths of 540 and 570 nm, respectively.

### Histological evaluation of aortic atherosclerotic lesions

Post-treatment, anesthesia was induced with 1% pentobarbital, and aorta tissue was carefully immersed and incubated in physiological saline before being fixed in 4% formalin. Subsequently, the tissue was dehydrated using sucrose solutions. Tissues were then embedded in freezing medium and sliced at 8 μm using a Thermo cryotome (Thermo Scientific, Waltham, MA, USA). Oil Red O-stained and hematoxylin-counterstained Sections. [10–12] were analyzed by microscopy (Leica CS2), and ImageJ was used to assess lesion dimensions.

### Statistical analysis

All evaluation was carried out using SPSS v20.0. For normally distributed data, one-way analysis of variance (ANOVA) was used to compare differences among multiple groups. When significant differences were identified, Tukey’s post hoc test was applied to determine specific pairwise differences. For non-normally distributed data, the Mann-Whitney U test was utilized to evaluate intergroup differences. Two-group comparisons applied Student’s t-test. Linear relationships between continuous variables were evaluated using Pearson correlation. Outcomes are represented as mean ± standard deviation (SD), with *P* < 0.05 indicating significance.

## Results

### Detection and characterization of genes (DEGs) associated with AS

**Fig. 1:**
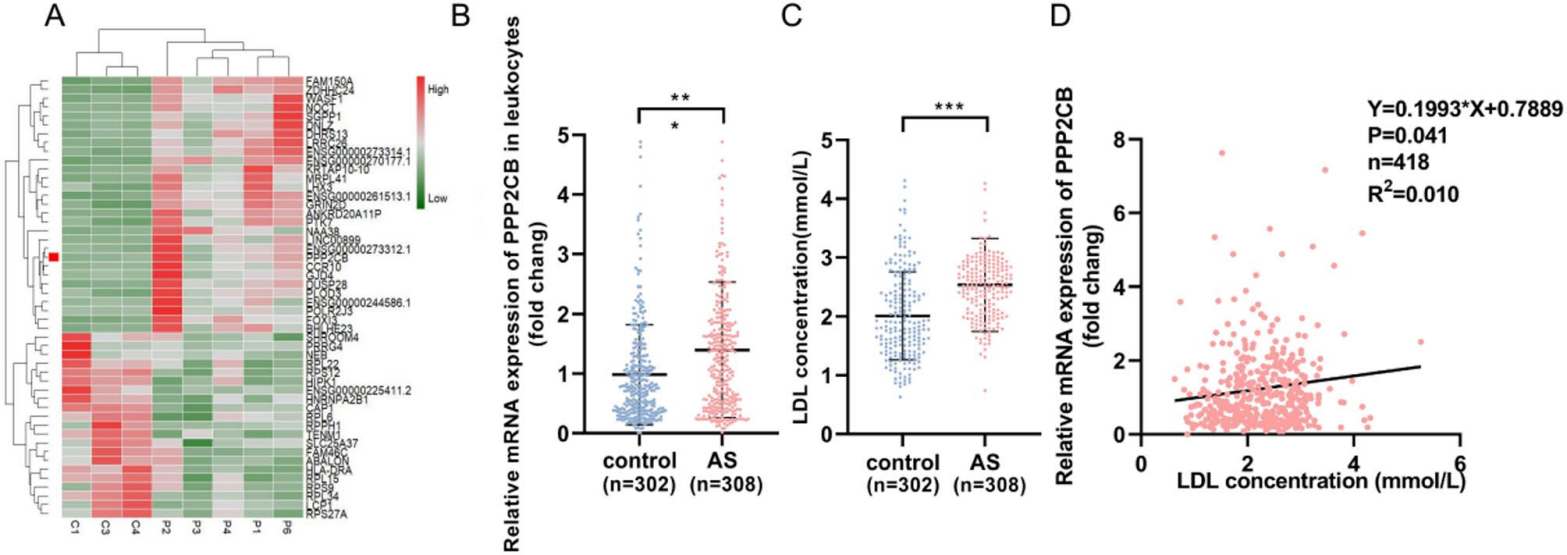
Detection and characterization of genes (DEGs) associated with AS. **(A)** The heatmap displays the mRNA expression in plasma exosomes from 5 STEMI patients and 3 controls, obtained through RNA-seq. Red indicates upregulated genes, while green indicates downregulation. The heat map highlights significant mRNA expression differences between STEMI patients and controls. **(B)** Comparison of PPP2CB mRNA expression levels in peripheral blood leukocytes across the control cohort (*n* = 302) versus the AS cohort (*n* = 308). Results show that PPP2CB expression is significantly higher in the AS group, suggesting a potential association with the pathophysiology of atherosclerosis. **(C)** Plasma LDL levels in both AS and control cohorts. The findings indicate a notable LDL increase in AS patients, implying greater susceptibility to cardiovascular events. **(D)** The correlation between PPP2CB expression in peripheral blood (*n* = 418) leukocytes and plasma LDL levels. The relationship between PPP2CB expression and LDL concentration was assessed using Pearson correlation analysis, yielding a *P*-value of 0.041 and a correlation coefficient (R) of 0.1. A positive correlation was observed, suggesting a potential link between PPP2CB expression and lipid metabolism. **P < 0.01, ***P < 0.001

In order to determine the different RNA expression profiles among the groups, transcriptome analysis of plasma exosomes from 5 STEMI patients and 3 controls was performed by RNA-seq. The heatmap displays the significantly differentially expressed mRNAs. There were 173 upregulated genes and 21 downregulated genes. PPP2CB exhibited significant differences between STEMI patients and controls (Fig. 1A). Subsequently, PPP2CB expression in peripheral blood leukocytes was evaluated via qRT-PCR, which showed a marked elevation in AS patients (*P <* 0.001, Fig. 1B, t-test). The plasma LDL levels in AS subjects were notably increased relative to the control population (*P <* 0.001, Fig. 1C, t-test). Correlation analysis confirmed a positive association between PPP2CB expression in peripheral blood leukocytes and LDL levels, as assessed using Pearson’s correlation coefficient (*P* = 0.041, *R* = 0.1, Fig. 1D). Further detailed information on clinical characteristics is presented in Supplementary Table S3.

### PPP2CB expression is upregulated in ApoE^−/−^ hyperlipidemic mouse atheromatous plaque and HS-induced hepatic cells

**Fig. 2:**
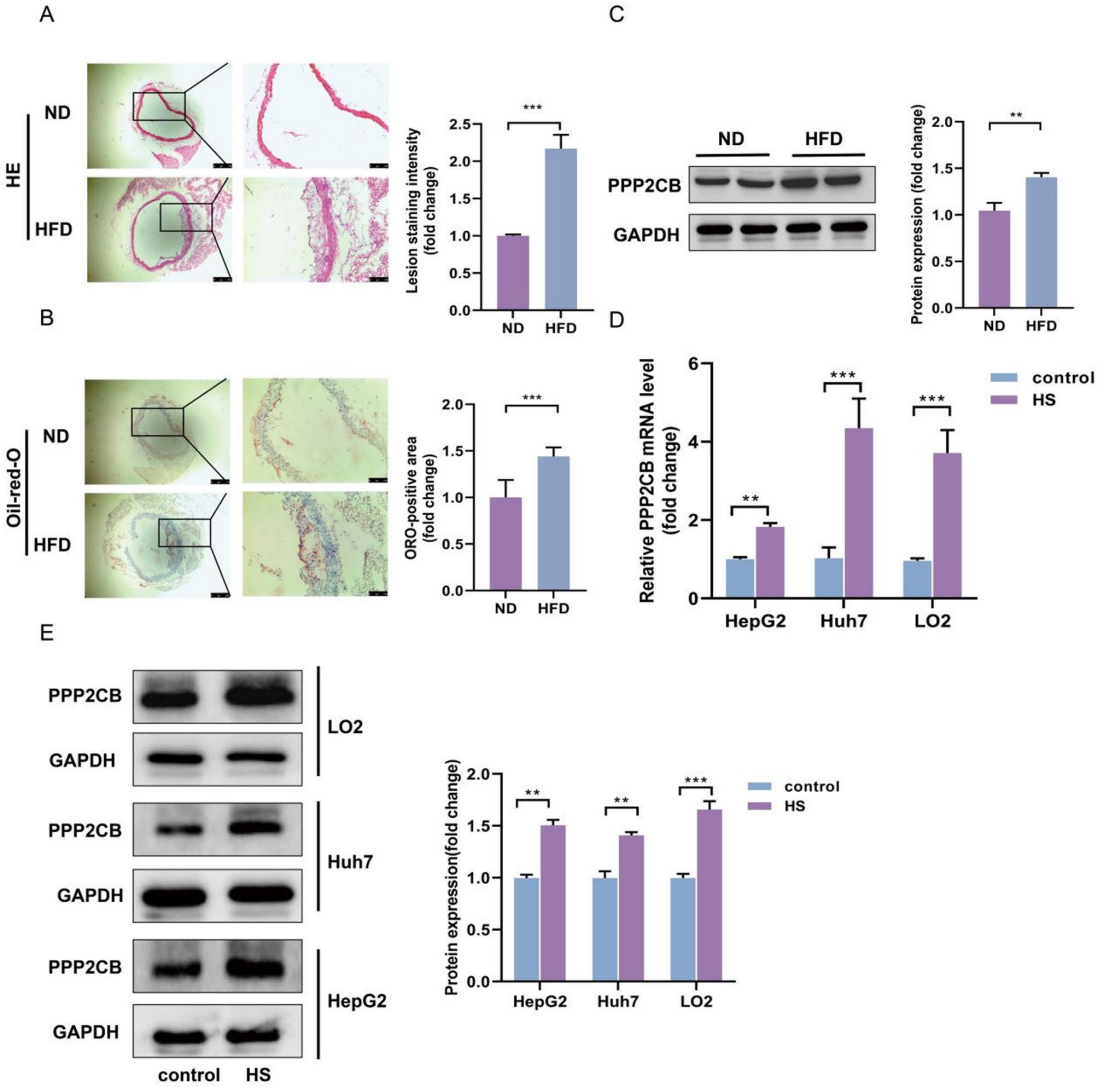
Analysis of PPP2CB expression in ApoE^−/−^ hyperlipidemic mouse plaques and HS-induced hepatic cells. **(A)** Heart tissue samples from three murine groups (*n* = 3 per mouse per group) were subjected to HE staining to assess plaque regions. HE staining was used to evaluate plaque size and morphological changes, confirming that HFD successfully induced atherosclerosis. The plaque burden was quantified as the ratio of plaque area to total vessel area using ImageJ, with values normalized to the control group and expressed as fold changes. **(B)** Heart tissue samples from three groups of mice were subjected to Oil Red O staining for detection of plaque area. This experiment evaluated the extent of lipid accumulation in the plaques, further supporting the development of the atherosclerotic model in HFD-fed mice. The plaque burden was quantified as the ratio of plaque area to total vessel area using ImageJ, and the values were calibrated against the control group and reported in terms of fold change. **(C)** PPP2CB protein levels in heart tissue plaques of ApoE^−/−^ mice exposed to either ND or HFD were analyzed by Western blotting. The results show higher PPP2CB protein expression in the HFD group, suggesting its potential involvement in the progression of atherosclerosis. **(D)** PPP2CB mRNA expression levels in HepG2, Huh7, and LO2 cells were determined by qRT-PCR after 24 h of HS treatment. This experiment aimed to evaluate whether HS treatment affects PPP2CB gene expression in different hepatic cell lines, serving as a potential marker for lipid metabolism and inflammation. **(E)** PPP2CB protein expressions levels in HepG2, Huh7, and LO2 cells were measured by Western blotting after 24 h of HS treatment. Immunoblot analysis revealed a significant upregulation in PPP2CB levels. **P < 0.01, and ***P < 0.001

To evaluate the expression pattern of PPP2CB within atheromatous plaques during the progression of atherosclerosis, atherosclerotic lesions were isolated from the heart tissues of ApoE^-/- mice fed a HFD for 14 weeks and compared with tissues from mice maintained on a ND. The results of HE staining and Oil Red O staining were analyzed for intergroup differences using Student’s t-test. The plaque area was larger in the heart tissue of ApoE^−/−^ mice on a HFD relative to ND-fed counterparts, indicating successful construction of the AS mice model (Fig. 2A and B). Additionally, Western blotting analysis revealed upregulation of PPP2CB expression in ApoE^−/−^ mice exposed to HFD relative to ND controls (*P <* 0.01, Fig. 2C).

Previous research revealed that individual metabolic variations are intricately interrelated, and changes in a single metabolic molecule are insufficient to accurately stratify disease risk [24]. HepG2, Huh7, and LO2 cells were treated with HS for 24 h. The results of qRT-PCR (Fig. 2D) and immunoblotting (Fig. 2E) showed that PPP2CB expression was significantly elevated after HS treatment.

### PPP2CB aggravates lipid deposition in hepatocytes

**Fig. 3:**
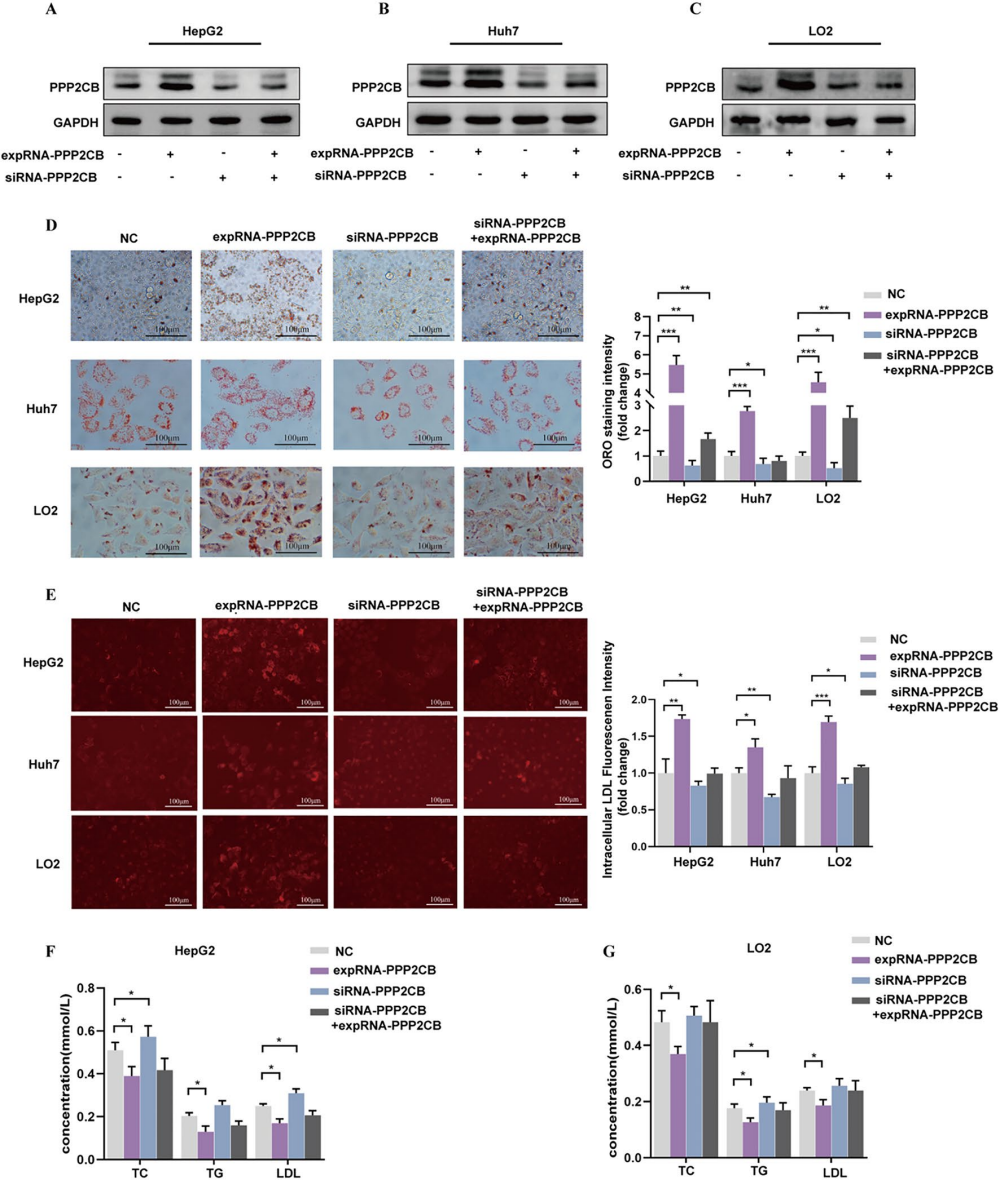
Analysis of intracellular lipid accumulation in hepatic cells following PPP2CB overexpression or silencing. **(A-C)** Western blotting results demonstrated successful modulation of PPP2CB expression in HepG2, Huh7, as well as LO2 cells through overexpression and silencing strategies, thereby enabling evaluation of its impact on lipid metabolism in these hepatic cell lines. **(D)** Oil Red O-based lipid visualization (scale bar = 100 μm) in HepG2, Huh7, and LO2 cells. The quantification of lipid droplets is shown on the right, expressed as fold changes relative to the NC group. Quantification of lipid droplets was performed to evaluate the extent of lipid accumulation in response to altered PPP2CB expression. **(E)** HepG2, Huh7, and LO2 cells were stimulated with fluorescence-labeled LDL (LDL-DyLight 550) for 3.5 h. Representative fluorescence images of intracellular LDL are shown (scale bar = 100 μm). The fluorescence intensity of intracellular LDL was quantified. The red fluorescence indicates the labeling of LDL. This experiment assessed the uptake of LDL, a key process in lipid metabolism. **(F)** At 24 h post-transfection, HepG2 cells were treated with 10% HS for 12 h. The concentrations of TC, TG as well as LDL in the supernatant were measured using an autoanalyzer to evaluate lipid secretion under altered PPP2CB expression. **(G)** The LO2 cells were treated as described in panel. The supernatant levels of TC, TG, as well as LDL were measured using autoanalyzer. This experiment provided additional insights into PPP2CB-mediated lipid handling in hepatocytes. *P < 0.05, **P < 0.01, and, ***P < 0.001

To comprehensively elucidate the function of PPP2CB in lipid regulation, PPP2CB was either overexpressed or silenced in HepG2, Huh7, and LO2 cell lines. A significant increase or decrease in PPP2CB levels was observed following transfection with expRNA-PPP2CB or siRNA-PPP2CB, respectively (Fig. 3A and C). Given the observed elevation of PPP2CB expression in HS-induced hepatic cells, lipid accumulation was subsequently evaluated using Oil Red O staining. The results revealed a significant increase in intracellular lipid deposition following transfection with expRNA-PPP2CB. In contrast, the transfected lipid buildup declined in the siRNA-PPP2CB cohort relative to the negative control cohort (Fig. 3D). An increase in intracellular LDL uptake was observed following expRNA-PPP2CB transfection (Fig. 3E). Additionally, PPP2CB overexpression in HepG2 and LO2 cells led to a marked reduction in the secretion of LDL, TC, TG and in the conditioned medium, which was reversed by PPP2CB knockdown (Fig. 3F and G).

### PPP2CB targets LOX-1 and enhances its expression

**Fig. 4:**
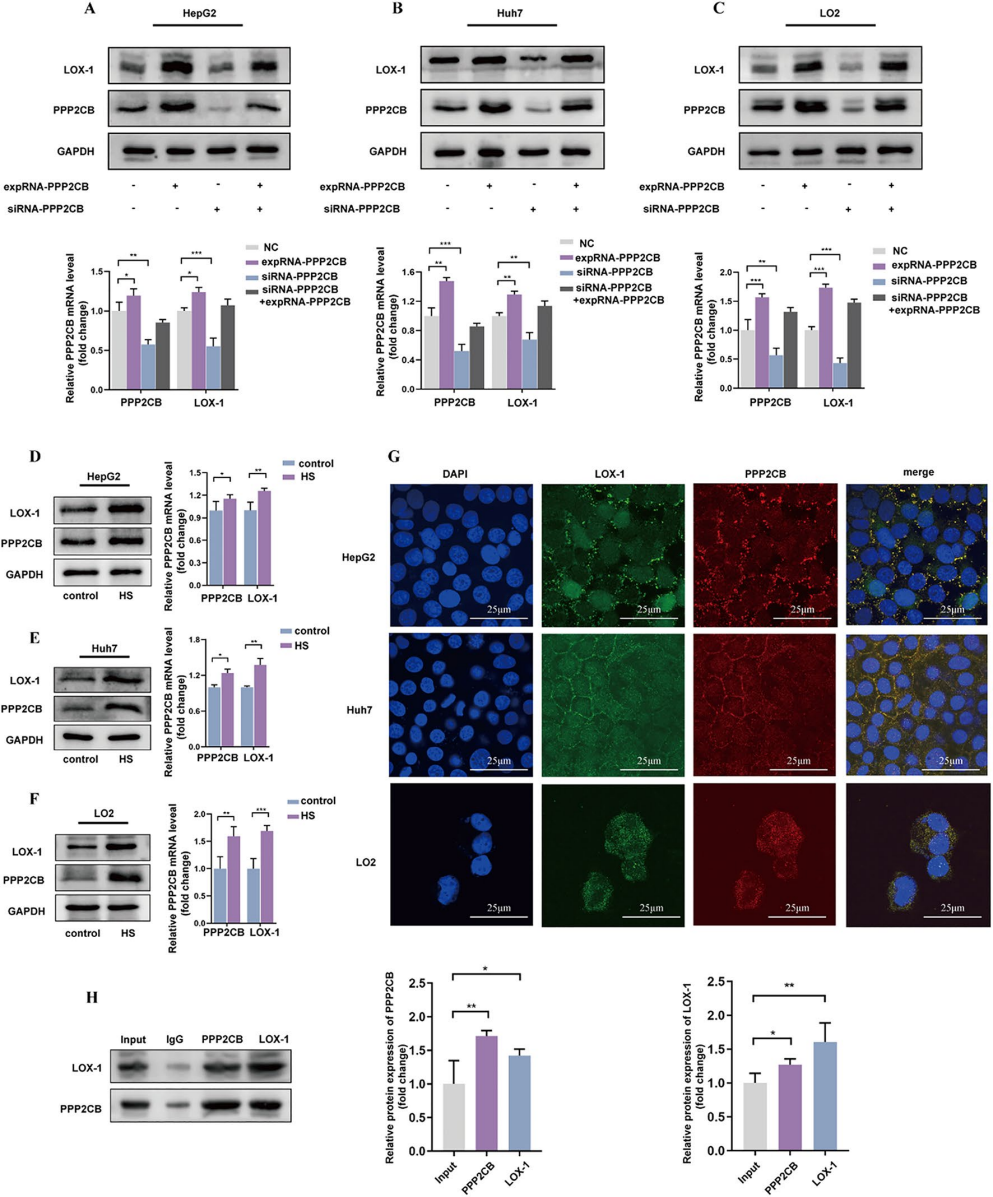
Analysis of the relationship between PPP2CB and LOX-1 in hepatic cells. **(A-C)** Cells underwent transfection negative control (NC), expRNA-PPP2CB (over-expression), or siRNA-PPP2CB (silencing), and then challenged with 10% HS for 24 h. Immunoblotting analysis and densitometric quantification were performed to measure the expression levels of PPP2CB and LOX-1. The results showed that overexpression of PPP2CB enhanced LOX-1 expression, while knockdown of PPP2CB reduced LOX-1 expression. **(D-F)** HepG2 (D), Huh7 (E), and LO2 (F) were challenged with 10% HS for 24 h. Western blotting analysis and densitometric quantification of PPP2CB alongside LOX-1 were performed. Findings demonstrated that HS treatment led to a synchronous upregulation of both PPP2CB and LOX-1 expression, further supporting their concurrent expression in hepatic cells. **(G)** HepG2, Huh7, and LO2 were treated as described in panels. (D-F) Illustrative immunofluorescence images of PPP2CB (red) and LOX-1 (green) are shown. Nuclei were stained using DAPI (blue). Scale bar = 25 μm. PPP2CB and LOX-1 colocalization in cells suggests that the two proteins may interact with each other. **(H)** Co-immunoprecipitation of PPP2CB and LOX-1 in LO2 cells challenged with 10% HS. A significant increase in LOX-1 protein level was detected within the PPP2CB antibody complex relative to both the IgG control and input groups, indicating a potential direct interaction between PPP2CB and LOX-1. *P < 0.05, **P < 0.01, and ***P < 0.001

Following knockdown and overexpression of PPP2CB, the cells were then stimulated with 10% HS for 24 h. PPP2CB overexpression significantly upregulated LOX-1 expression, while its knockdown reduced LOX-1 expression (Fig. 4A and C). To further confirm these findings, hepatic cells were treated with HS, which led to synchronous upregulation of PPP2CB and LOX-1 (Fig. 4D and F).

To investigate whether PPP2CB interacts with LOX-1, cells were exposed to 10% HS and subjected to immunofluorescence co-staining. Colocalization of PPP2CB and LOX-1 was observed in all three hepatic cell lines (Fig. 4G). Furthermore, co-immunoprecipitation experiments indicated a notable enrichment of LOX-1 within the PPP2CB antibody complex compared to the IgG control and input samples (Fig. 4H). These results strongly suggest a physical interaction between PPP2CB and LOX-1.

### LOX-1 promotes lipid accumulation by activating MAPK/ERK signaling axis

**Fig. 5:**
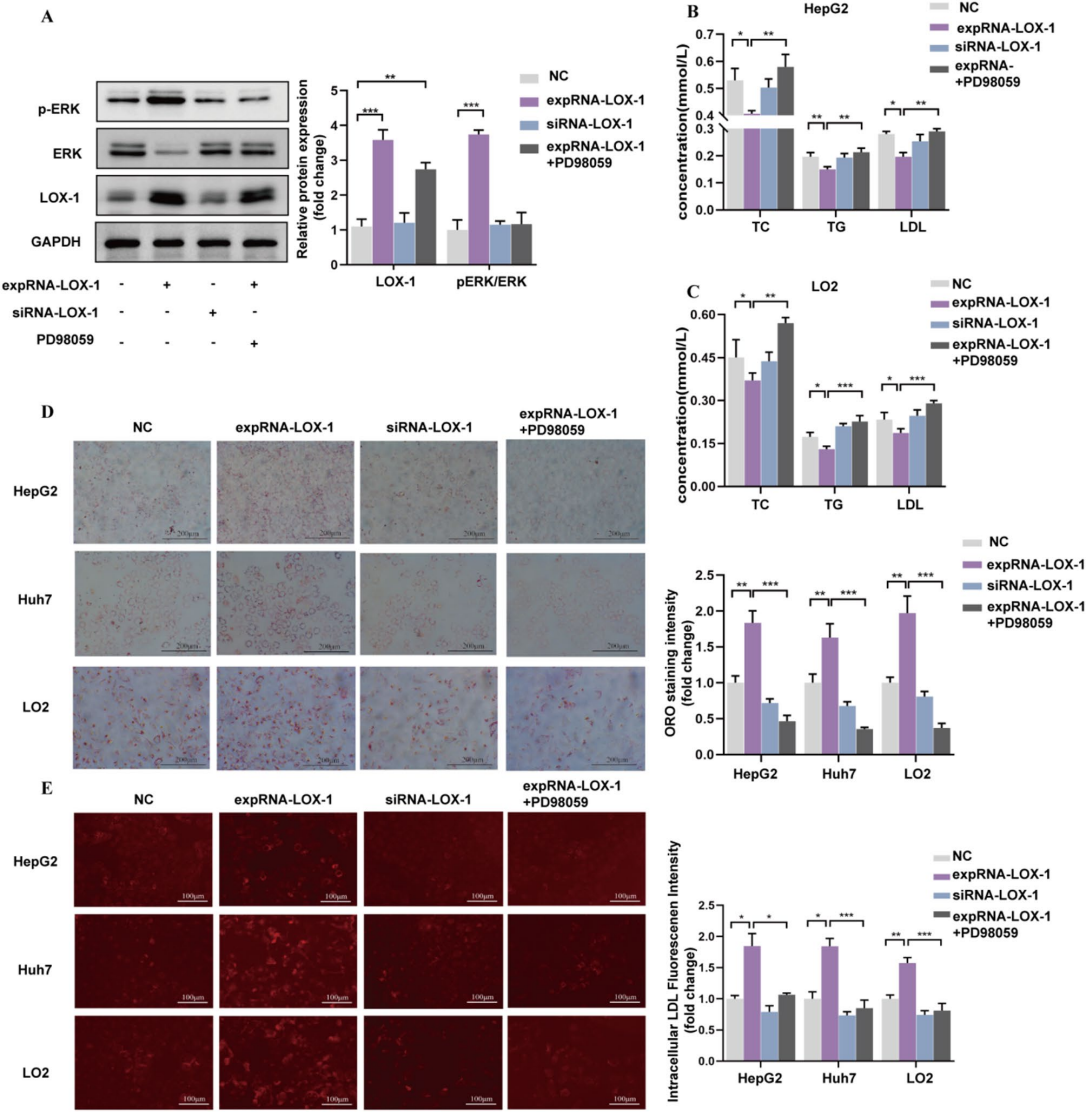
LOX-1 promotes lipid accumulation through MAPK/ERK pathway activation. **(A)** LO2 cells were transfected with negative control (NC), expRNA-LOX-1, siRNA-LOX-1, or expRNA-LOX-1 combined with PD98059. Western blotting assessed ERK, p-ERK, and LOX-1 levels, followed by densitometric quantification to evaluate MAPK/ERK signaling activation and the impact of LOX-1 modulation. **(B)** After transfection for 24 h, HepG2 cells were treated with 10% HS for 12 h, and the supernatant was collected. The concentrations of TC, TG, and LDL were measured using an autoanalyzer to assess lipid secretion in response to LOX-1 overexpression and activation of the MAPK/ERK pathway. **(C)** LO2 cells were treated as described in panel (B), and lipid concentrations in the supernatant were measured to further investigate the effect of LOX-1 and the MAPK/ERK pathway on lipid metabolism in different hepatic cell lines. **(D)** Illustrative Oil Red O-stained images (scale bar = 200 μm) accompanied by quantification of lipid droplets in HepG2, Huh7, and LO2 cells. This experiment was conducted to assess intracellular lipid accumulation in response to LOX-1 overexpression and MAPK/ERK pathway activation. (**E**) HepG2, Huh7, and LO2 cells were incubated with LDL-DyLight 550 for 3.5 h. Fluorescence-based imaging results and quantification of LDL accumulation within HepG2, Huh7, and LO2 cells are shown (scale bar = 100 μm). The LDL was labeled with red fluorescence. The results demonstrate that LOX-1 overexpression enhances LDL uptake, which is linked to lipid accumulation in these cells. *P < 0.05, **P < 0.01, and ***P < 0.001

To investigate the downstream cascades triggered by LOX-1, key components of the MAPK/ERK signaling cascade were examined via Western blotting. Overexpression of LOX-1 (expRNA-LOX-1) reduced total ERK levels while increasing phosphorylated ERK (p-ERK), consistent with MAPK/ERK signaling activation (Fig. 5A). Treatment with the ERK inhibitor PD98059 (20 µM) effectively blocked this activation, as evidenced by reduced p-ERK expression. In addition, LOX-1 overexpression led to a significant decrease in TC, TG, and LDL in the conditioned media of HepG2 and LO2 cells. This reduction was reversed by PD98059 treatment (Fig. 5B and C). To further assess the impact of PPP2CB and ERK inhibition on lipid accumulation, Oil Red O staining was performed following transfection with expRNA-PPP2CB or siRNA-PPP2CB, with or without PD98059 (Fig. 5D). The results showed that PD98059 mitigated LOX-1-induced intracellular lipid accumulation. Moreover, fluorescence-based LDL uptake assays revealed that LOX-1 overexpression significantly increased intracellular LDL uptake, which was markedly reduced upon PD98059 treatment (Fig. 5E). These findings suggest that LOX-1 promotes hepatic lipid accumulation through MAPK/ERK activation, while pharmacological inhibition of ERK can effectively attenuate this effect.

### Overexpression of PPP2CB and LOX-1 significantly activates the MAPK/ERK signaling pathway

**Fig. 6:**
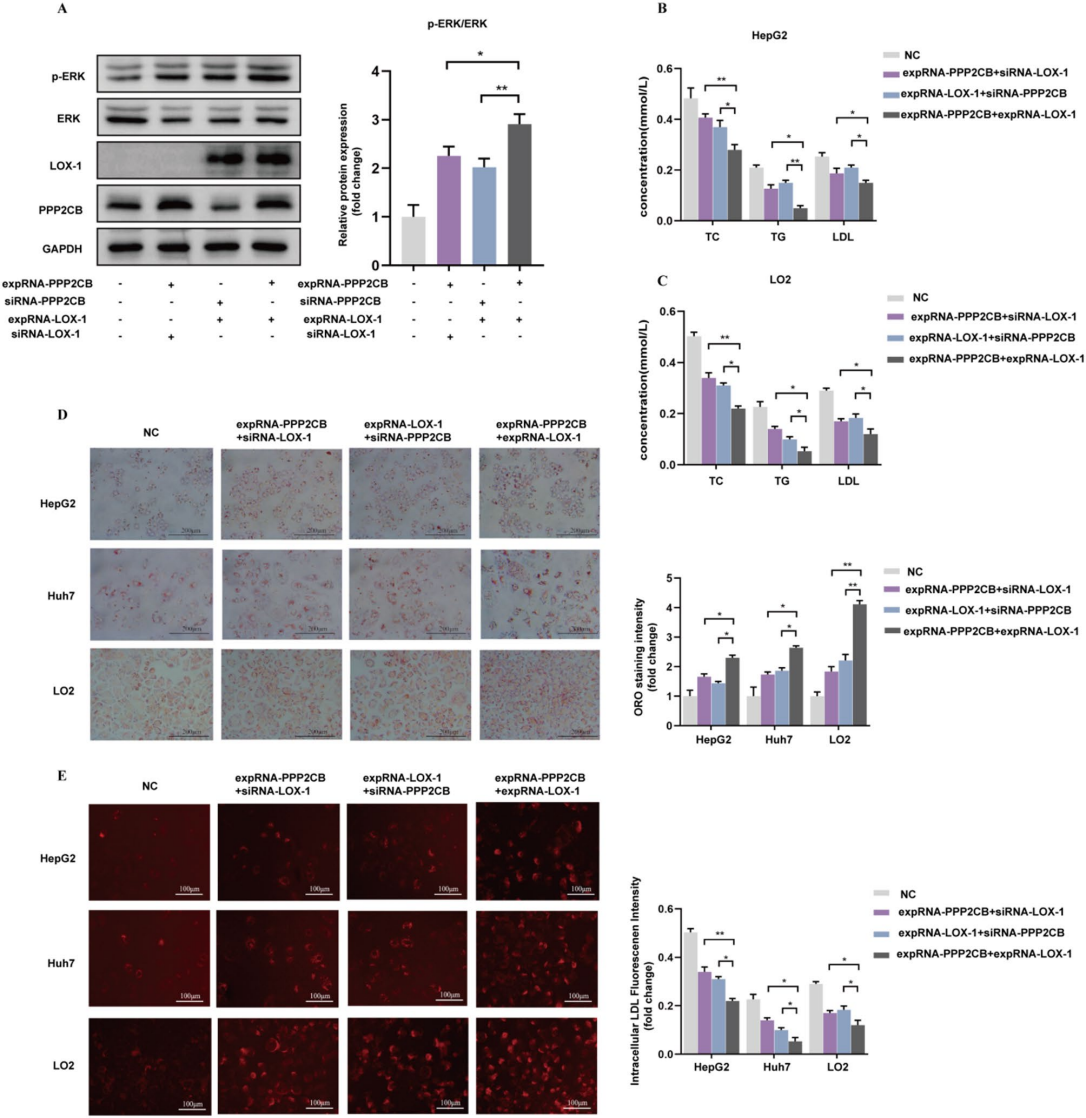
Overexpression of PPP2CB and LOX-1 significantly activates the MAPK/ERK signaling pathway. **(A)** LO2 cells were transfected with negative control (NC), expRNA-PPP2CB, siRNA-PPP2CB, expRNA-LOX-1, and siRNA-LOX-1 respectively. Western blotting was performed to confirm the protein expression levels of PPP2CB, LOX-1, p-ERK, and ERK. The ratio of p-ERK/ERK was quantified using ImageJ to assess MAPK/ERK pathway. **(B)** At 24 h post-transfection, HepG2 cells were treated with 10% HS for 12 h. TC, TG, and LDL concentrations in the culture supernatant were measured using an autoanalyzer. **(C)** LO2 cells were treated under the same conditions in panel (B), and supernatant lipid levels were assessed to validate the effects of PPP2CB and LOX-1 overexpression in a different hepatic cell line. **(D)** Oil Red O staining images (scale bar = 200 μm) and lipid droplet quantification are shown for HepG2, Huh7, and LO2 cells. This experiment was designed to assess the intracellular lipid buildup within cells overexpressing PPP2CB and LOX-1, as well as to determine the impact of MAPK/ERK activation on lipid storage. **(E)** HepG2, Huh7, and LO2 cells were incubated with LDL (LDL-DyLight 550) for 3.5 h. Red fluorescence indicates LDL uptake. Representative fluorescence images and quantification of intracellular LDL accumulation are shown (scale bar = 100 μm); *P < 0.05, **P < 0.01, and ***P < 0.001

Based on this premise, the study investigated whether PPP2CB-induced intracellular lipid accumulation occurs through its interaction with LOX-1 and ensuing activation of the MAPK/ERK signaling cascade. Co-transfection with expRNA-PPP2CB and expRNA-LOX-1 significantly enhanced MAPK/ERK signaling pathway activation compared to transfection with either PPP2CB or LOX-1 alone (Fig. 6A). In addition, co-overexpressing of PPP2CB and LOX-1 in HepG2 and LO2 cells led to a marked reduction in TC, TG, and LDL levels in the conditioned medium (Fig. 6B and C). This co-expression also significantly promoted intracellular lipid accumulation (Fig. 6C). Overexpression of PPP2CB or LOX-1 alone increased intracellular LDL uptake, while their co-expression further enhanced this effect (Fig. 6D and E).

## Discussion

Dyslipidemia, which is characterized by elevated levels of TC, LDL-C, TG, or low levels of HDL cholesterol, is a major risk factor for AS [26]. The regulation of dyslipidemia involves a complex process of intracellular pathways, including post-translational phosphorylation. PP2A, an essential enzyme targeting serine/threonine sites, is indispensable for this regulation. Macrophage-specific loss of PP2A has been found to elevate p38 phosphorylation, resulting in the upregulation of the scavenger receptor cluster of differentiation 36 (CD36). This, in turn, promotes the uptake of lipoproteins and triggers the development of AS [14]. Additionally, Xu et al. reported that PP2A mitigated HUVEC dysfunction and ox-LDL-induced inflammation through modulation of the LOX-1/ROS/MAPK signaling axis, demonstrating the anti-inflammatory effect of PP2A during AS formation [27].

PP2A is composed of three subunits: the catalytic C, scaffolding A, and regulatory B [28]. The C-subunit serves as an essential factor for assembling the PP2A dimer and holoenzyme complex in cells [29]. There are two isoforms of the catalytic core of PP2A, PPP2CA and PPP2CB. Accumulating evidence suggests that PPP2CB participates in various biological processes, including apoptosis and metabolism [30]. In the present study, elevated PPP2CB gene expression was observed in peripheral blood cells from patients with atherosclerosis, atheromatous plaques from atherosclerotic mice, and hyperlipidemic hepatic cells in vitro, compared to normal controls. These findings indicate that the various isoforms of PP2A may have distinct roles in disease pathogenesis. Moreover, ApoE is involved in multiple aspects of lipoprotein homeostasis. It facilitates the endocytic clearance of plasma lipoproteins, particularly very low-density lipoprotein (VLDL) and remnant lipoproteins, through interaction with its receptors [31]. A potential regulatory association between dyslipidemia-driven PPP2CB upregulation and ApoE was indicated. Nevertheless, further research efforts are warranted to uncover the expression patterns as well as functional implications of PPP2CB at the molecular, cellular, and tissue levels in cardiovascular disease.

Dysregulation of endothelial function contributes to the onset of pathological changes in cardiovascular disease development. Dysregulation is induced by oxidized LDL binding to LOX-1. LOX-1 activity suppresses nitric oxide release via oxidative stress and phosphorylation of endothelial nitric oxide synthase (eNOS) [32, 33]. Pirillo et al. reported that LOX-1 was nearly undetectable in physiological contexts [34]. However, it becomes upregulated in response to various proatherogenic stimuli and can be detected in both animal and human atherosclerotic lesions. In this study, upregulation of PPP2CB under HS treatment was accompanied by LOX-1 overexpression. Moreover, their direct interaction was confirmed through co-immunoprecipitation and co-localization analyses. Additionally, molecular docking was performed using the protein docking tool GRAMM-X to investigate the interaction between the ligand (PPP2CB) and the receptor (LOX-1). The highest-ranked model was analyzed in the PDBePISA database, revealing an interface area of 931.3 (Å2) and a free energy of -13.5 (ΔiG kcal/mol) in this docking method. These results indicate a high binding capacity between PP2CB and LOX-1 (supplementary Fig. S1). A previous study reported that LOX-1 required protein interactions for intracellular signaling since it did not possess any recognizable enzymatic or catalytic activity in its cytoplasmic tail [35]. Two potential mechanisms are proposed: (a) PPP2CB interacts with LOX-1 through G9a-GLP. The phosphatase PPP2CB can dephosphorylate T1045 during late mitosis to activate G9a, thereby promoting mitotic exit [36]. G9a predominantly forms a heteromeric complex known as G9a-GLP, which functions as a histone lysine methyltransferase in vivo [37]. GLP can regulate ROS production through LOX-1 [38], and enhancing GLP activation was found to reduce LOX-1 levels and facilitate acetylcholine-triggered vasodilation in aortas [39]; (b) PPP2CB recognizes the intron region of LOX-1 that contains the carbohydrate-recognition domain. Deletion of PPP2CB specifically enhances T-cell activation induced by phorbol myristate acetate plus ionomycin and increases cluster of differentiation 69 (CD69) expression [18]. The first intron in the carbohydrate-recognition domain of LOX-1, which separates the cytoplasmic tail from the transmembrane domain, is positioned similarly to that found in CD69 genes [40]. However, the precise mechanism by which PPP2CB interacts with LOX-1 remains incompletely understood.

Studies have shown that ox-LDL activates MAPKs through LOX-1-mediated endocytosis in vascular endothelial cells, leading to increased levels of monocyte chemotactic protein 1 (MCP-1) and vascular adhesion molecules (ICAM-1) [41]. The activation of ERK is crucial for adipogenesis and mitotic clonal expansion [40]. Sandra et al. conducted comprehensive lipidomic analyses and found that disruption of the MEK5/ERK5 axis affected various lipid metabolism pathways, such as the mevalonate route mediating cholesterol production [42]. Consistent with the finding observed in hepatic cells, Rusan et al. reported that ox-LDL stimulation led to significant accumulation of ox-LDL and rapid upregulation of LOX-1 in HUVECs. These effects were abolished upon the inhibition of ERK1/2, p38 MAPK, or AP-1 signaling pathways [43]. These studies suggest that the LOX-1/MAPK/ERK pathway may regulate a range of target genes, including MCP-1, matrix metalloproteinases (MMP-1, -3, -9), ICAM-1, and vascular cell adhesion molecule (VCAM-1), which play key roles in inflammation, cellular adhesion and matrix degradation [32]. Furthermore, the LOX-1/MAPK/ERK pathway may also upregulate Nox2 and Nox4, promoting ROS generation [44], which exacerbates endothelial damage and redox imbalance. Based on these observations, it can be inferred that lipids activate LOX-1, subsequently triggering the MAPK/ERK cascade, forming a feed-forward regulatory loop in both hepatic cells and arterial endothelial cells that perpetuates lipid imbalance.

Traditional indicators including c-reactive protein (CRP) and LDL-C are widely used to assess inflammation and lipid accumulation in AS. However, the present findings suggest that PPP2CB may provide additional mechanistic insights beyond these conventional markers. Unlike CRP [45], which primarily reflects systemic inflammation, and LDL-C [46], which indicates circulating lipid levels, PPP2CB is involved in intracellular signaling pathways (LOX-1/MAPK/ERK) that regulate lipid metabolism and vascular inflammation. This suggests that PPP2CB may play an active regulatory role in AS progression rather than serving merely as a risk indicator.

Furthermore, exosomal PPP2CB detection presents a potential non-invasive and stable biomarker for AS risk assessment and disease monitoring. While CRP and LDL-C have well-established clinical applications, PPP2CB’s diagnostic and prognostic value requires further validation in larger patient cohorts. Future studies should focus on evaluating PPP2CB’s predictive potential and its utility in combination with existing biomarkers to enhance AS risk stratification and disease surveillance.

In this study, female ApoE⁻/⁻ mice were used as the animal model to investigate the influence of sex on the progression of atherosclerosis. Although male ApoE⁻/⁻ mice are more commonly used, recent studies have highlighted that female mice may develop more severe atherosclerotic lesions under high-fat diet conditions, potentially due to differences in sex hormones such as estrogen and sex chromosome-linked gene expression [47]. In this study, the observed increases in plaque burden and lipid accumulation in HFD-fed female mice may partially reflect this sex-specific vulnerability. Estrogen has been shown to modulate lipid metabolism and vascular inflammation through multiple pathways, including sterol regulatory element-binding protein (SREBP) cascades [24]. These factors may have amplified the PPP2CB-mediated activation of the LOX-1/MAPK/ERK axis. Therefore, the inclusion of female mice adds a biologically relevant dimension to the study and underscores the need for future investigations to incorporate both sexes in order to elucidate sex-specific regulatory mechanisms in atherosclerosis.

### Study Strengths and Limitations

#### Strengths

The present work pioneers efforts to systematically analyze the contribution of PPP2CB within the context of atherosclerosis-related lipid metabolism using a comprehensive approach that integrates clinical data, animal models, and cellular experiments. The study design included a large cohort of human participants (*n* = 610), which enhances the generalizability of the clinical findings. Furthermore, the use of hyperlipidemic ApoE⁻/⁻ mice and multiple hepatic cell lines (HepG2, Huh7, LO2) allowed for a robust validation of PPP2CB function in both in vivo and in vitro settings. Mechanistically, the study uncovered a novel interaction between PPP2CB and LOX-1 and demonstrated the downstream activation of the MAPK/ERK pathway, contributing new insights into the intracellular regulatory network of dyslipidemia-induced atherosclerosis. Additionally, the potential application of exosomal PPP2CB as a non-invasive biomarker for AS was preliminarily established, opening a new avenue for clinical diagnosis and risk stratification.

### Limitations

This research presents some limitations. Initially, the effects of PPP2CB on lipid efflux-related proteins (such as ATP-Binding Cassette Transporter A1 (ABCA1) and SR-BI) were not investigated, which limits the understanding of its role in the broader context of hepatic lipid homeostasis. Second, while PPP2CB’s effect on early to mid-stage atherosclerosis was assessed, the 12-week high-fat diet model did not capture its potential influence on late-stage pathological features such as fibrosis and plaque rupture. Third, the study did not employ PPP2CB knockout or transgenic overexpression animal models, which constrains the ability to confirm causal relationships. Fourth, potential confounding variables, including patient medication history, comorbidities, and lifestyle factors, were not fully accounted for in the clinical analysis. Lastly, the experimental focus was limited to hepatocytes, and further validation in other atherosclerosis-related cells, including endothelial and macrophage lineages, is warranted to generalize the findings. In conclusion, the role of PPP2CB in lipid metabolism and vascular inflammation requires further investigation to better assess its potential as an AS biomarker and therapeutic target.

## Conclusion

PPP2CB was found to promote lipid accumulation and LDL uptake via LOX-1/MAPK/ERK signaling, contributing to atherosclerosis progression. Clinically, PPP2CB holds promise as both a diagnostic indicator and intervention candidate, offering potential to improve risk stratification and lipid-related vascular intervention. Measuring exosomal PPP2CB could complement conventional markers like LDL-C and CRP. Future studies should validate its clinical utility and explore PPP2CB-targeted therapies to support individualized atherosclerosis management.

## Electronic supplementary material

Below is the link to the electronic supplementary material.

Supplementary Material 1

Supplementary Material 2

Supplementary Material 3

Supplementary Material 4

## Data Availability

The data used to support the findings of this study are available from the corresponding author upon request.

## Abbreviations

AS: Atherosclerosis
PPP2CB: Protein phosphatase 2 catalytic subunit beta
LDL: Low-density lipoprotein
ox-LDL: Oxidized LDL
PP2A: Phosphatase 2A
STEMI: ST-segment elevation myocardial infarction
ND: Normal chow diet
HFD: High-fat diet
HS: Hyperlipidemic serum
TC: Total cholesterol
LDL-C: Low-density lipoprotein cholesterol
qRT-PCR: Quantitative real-time PCR
HDL: High-density lipoprotein
TG: Triglyceride
SD: Standard deviation
NC: Negative control
