# Supplementary material for: PPP2CB aggravates atherosclerosis-related dyslipidemia via LOX-1/MAPK/ERK signaling pathway
Source: Lipids Health Dis. 2025 Jul 3;24:229. doi: 10.1186/s12944-025-02647-x (PMC12224689; doi:10.1186/s12944-025-02647-x)
Supplement: Supplementary file 1 — Supplementary Material 1 [file 12944_2025_2647_MOESM1_ESM.pptx]

## Slide 1
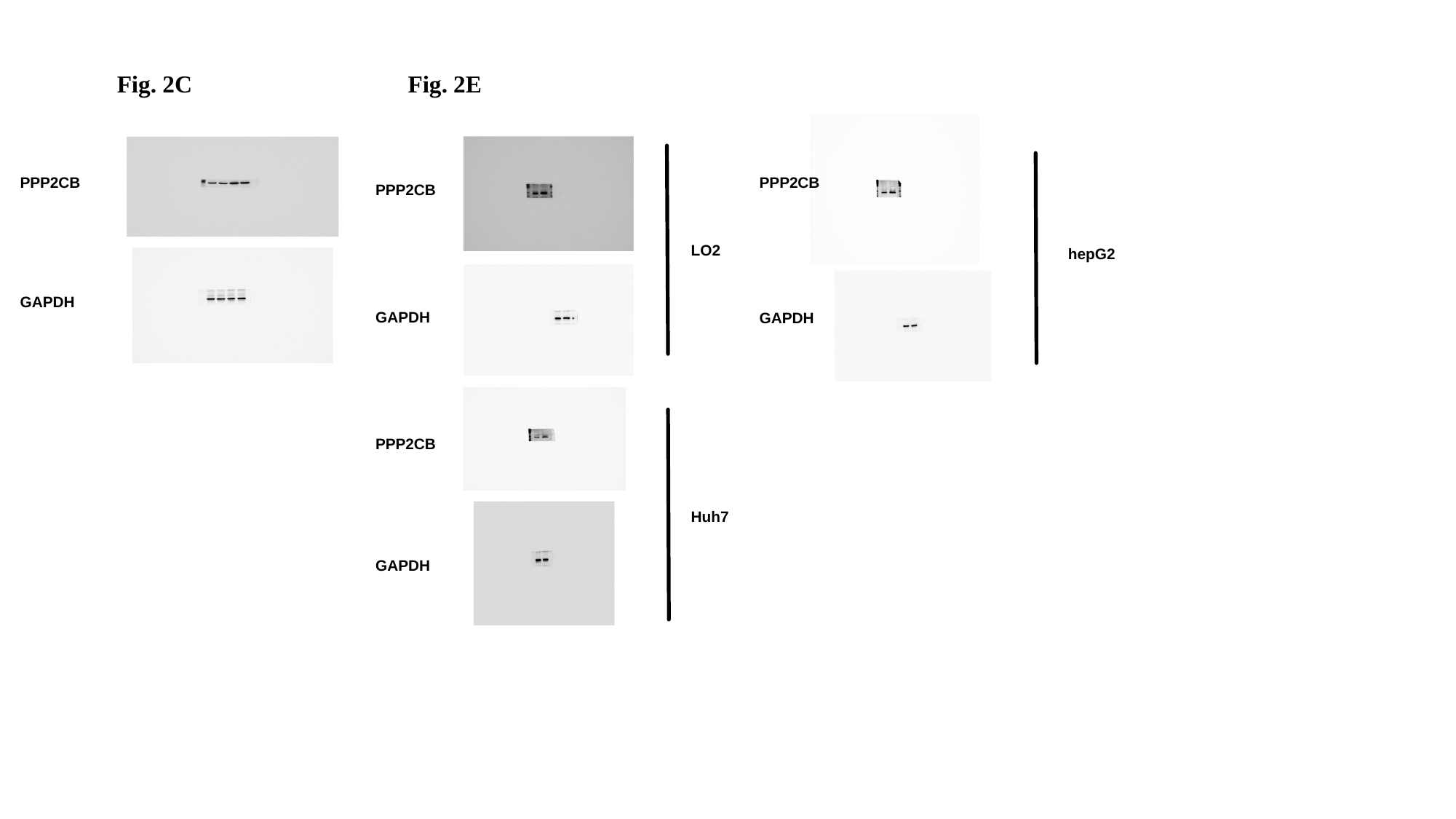

Fig. 2C
Fig. 2E
PPP2CB
PPP2CB
PPP2CB
LO2
hepG2
GAPDH
GAPDH
GAPDH
PPP2CB
Huh7
GAPDH

## Slide 2
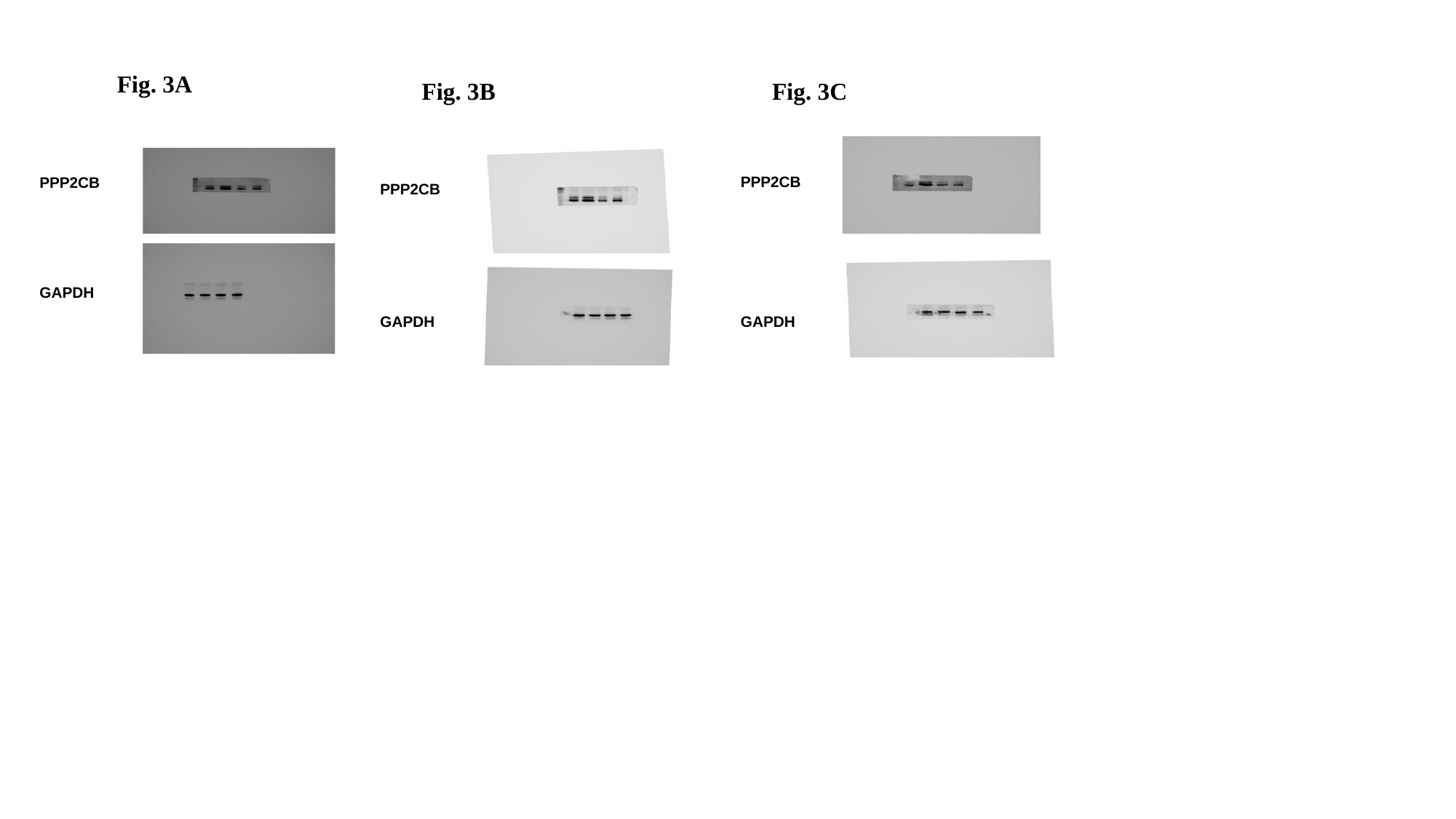

Fig. 3A
Fig. 3B
Fig. 3C
PPP2CB
PPP2CB
PPP2CB
GAPDH
GAPDH
GAPDH

## Slide 3
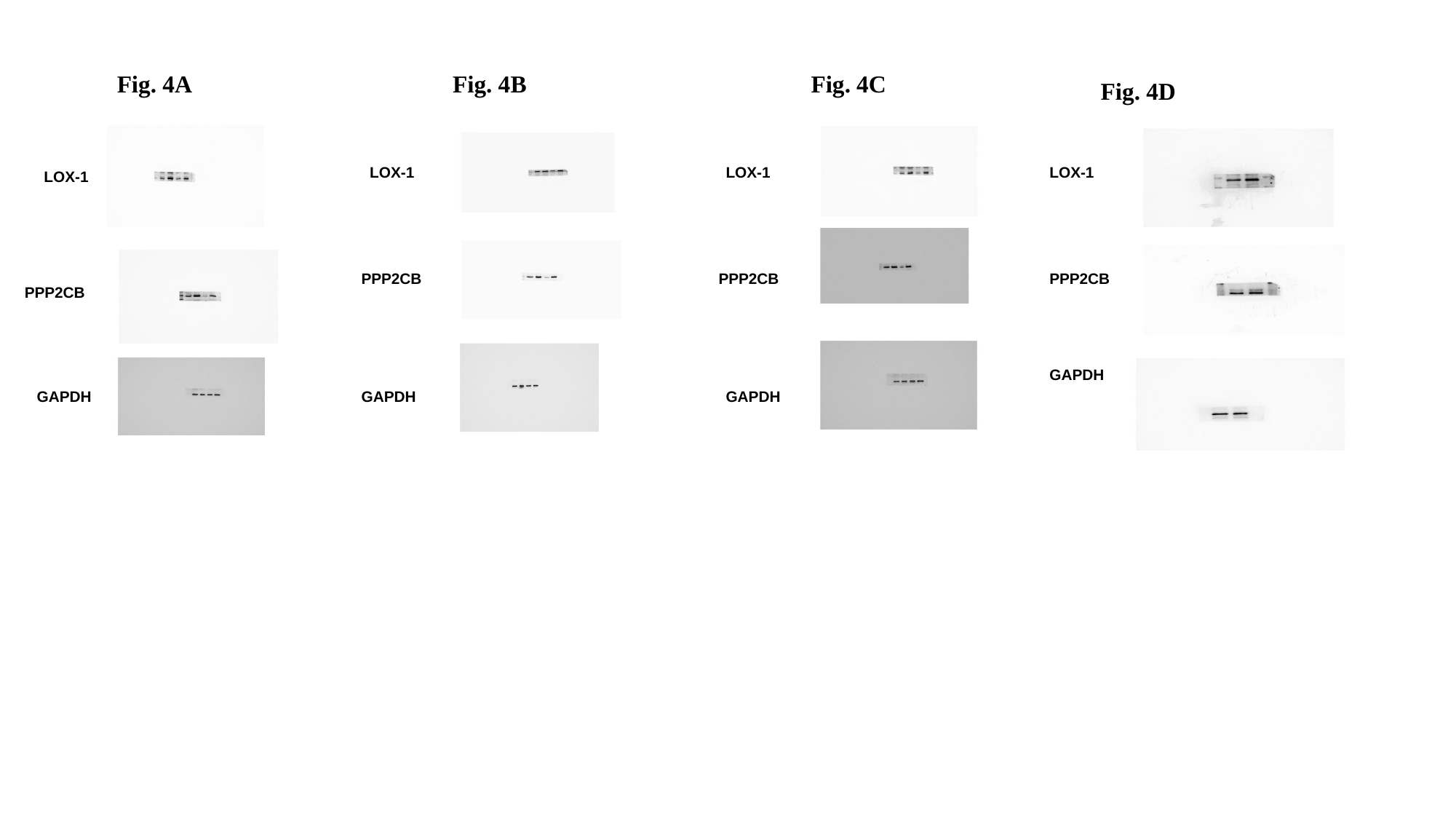

Fig. 4A
Fig. 4B
Fig. 4C
Fig. 4D
LOX-1
LOX-1
LOX-1
LOX-1
PPP2CB
PPP2CB
PPP2CB
PPP2CB
GAPDH
GAPDH
GAPDH
GAPDH

## Slide 4
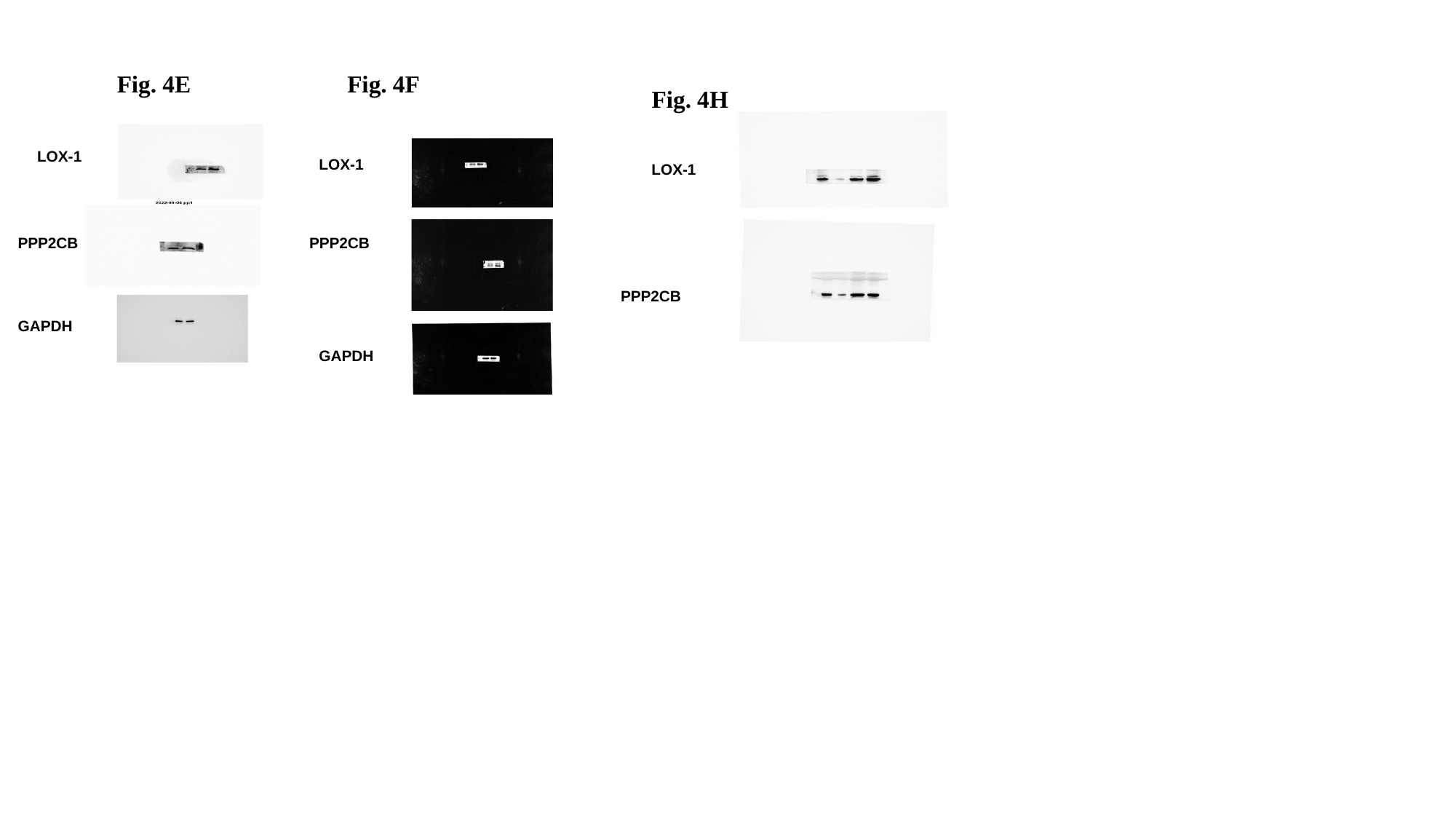

Fig. 4E
Fig. 4F
Fig. 4H
LOX-1
LOX-1
LOX-1
PPP2CB
PPP2CB
PPP2CB
GAPDH
GAPDH

## Slide 5
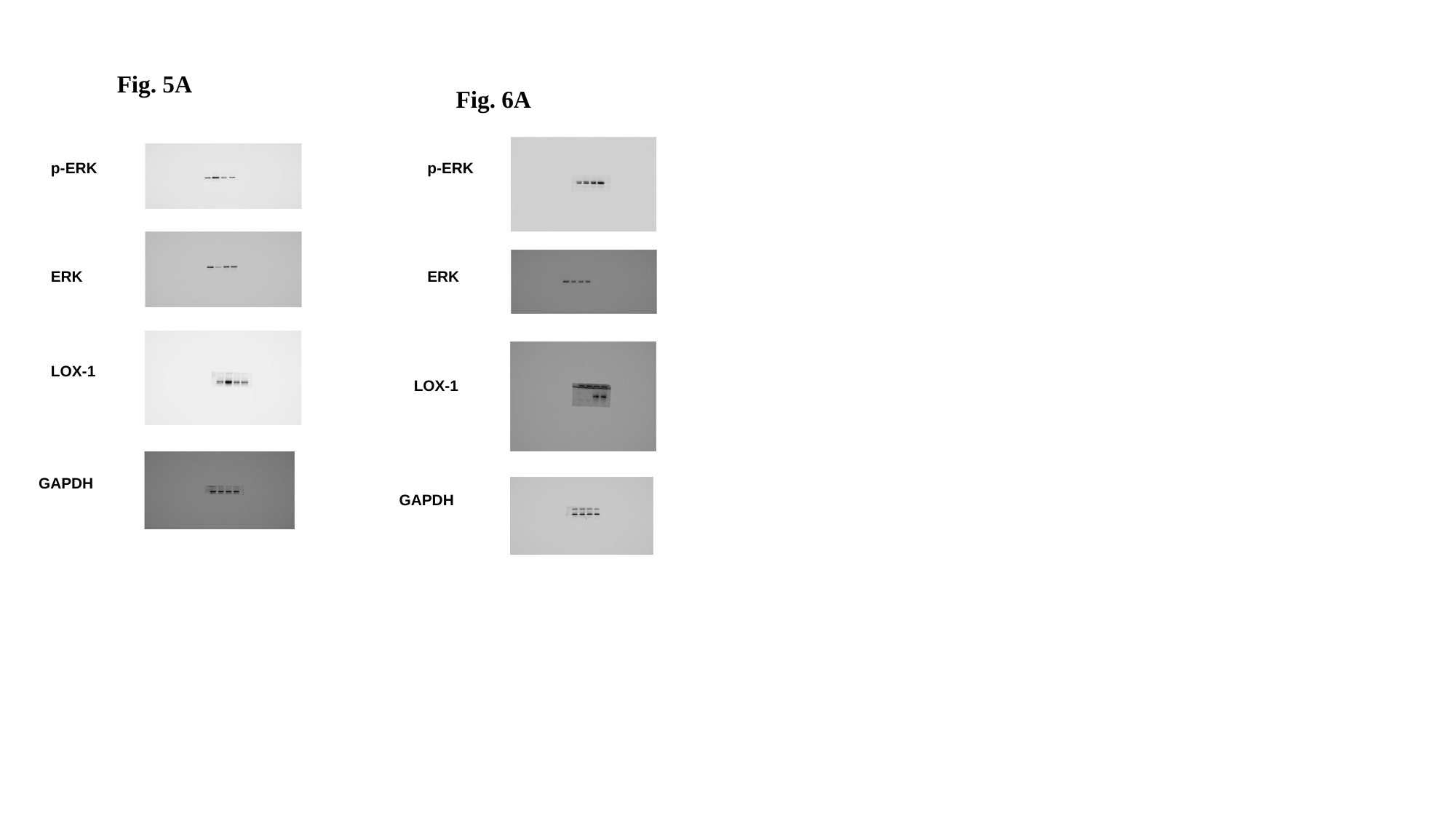

Fig. 5A
Fig. 6A
p-ERK
p-ERK
ERK
ERK
LOX-1
LOX-1
GAPDH
GAPDH
