# Supplementary material for: PPP2CB aggravates atherosclerosis-related dyslipidemia via LOX-1/MAPK/ERK signaling pathway
Source: Lipids Health Dis. 2025 Jul 3;24:229. doi: 10.1186/s12944-025-02647-x (PMC12224689; doi:10.1186/s12944-025-02647-x)
Supplement: Supplementary file 4 — Supplementary Material 4 [file 12944_2025_2647_MOESM4_ESM.pdf]

www.bio-medresearch.com

CERTIFICATE OF EDITING

Manuscript Title:

PPP2CB aggravates atherosclerosis-related dyslipidemia via LOX-1/MAPK/ERK signaling pathway

Author(s): He An

Date Issued: June.12, 2025

This document certifies that the abovementioned manuscript was edited and proofread by BIO MED RESEARCH LIMITED. These processes were executed to ensure that the manuscript is free of such English language errors as (but not limited to) those of clarity, coherence, diction, grammar, punctuation, spelling, and syntax.

Bio Med Research Limited

Issued by:

BIO MED RESEARCH LIMITED
Unit 04-05, 16th Floor,
The Broadway No. 54-62 Lockhart
Road, Wanchai, Hong Kong

Disclaimer: The intent of the author' s message has been preserved during the editing process. The author is free to accept or reject our changes in the document after reviewing our editing. This certificate has been awarded at the time of sharing the final edited version (full file or sections of the file) with the author. BIO MED RESEARCH LIMITED does not bear any responsibility for any alterations done by the author to the edited document post June.12, 2025.
